# Adenovirus Infection and Rhabdomyolysis as a Cause of Acute Liver Failure in a Healthy Collegiate Football Athlete: A Case Report and Proposed Return to Play Protocol for Rhabdomyolysis

**DOI:** 10.7759/cureus.14510

**Published:** 2021-04-15

**Authors:** Calvin E Hwang, Gordon Matheson, Jennifer Baine

**Affiliations:** 1 Orthopaedic Surgery, Stanford University, Stanford, USA; 2 Sports Medicine, Stanford University, Stanford, USA

**Keywords:** exertional rhabdomyolysis, adenovirus, sports medicine, liver failure, american football

## Abstract

Adenovirus is a common cause of upper respiratory and gastrointestinal tract infections. Though cases of significant organ failure and death have been reported in young children and immunocompromised individuals, adenovirus infections in healthy individuals are typically self-limiting without significant morbidity or mortality. Exertional rhabdomyolysis is a pathologic condition resulting from repetitive, excessive, or prolonged exercise, often in a hot environment, leading to acute muscle injury, renal injury and, rarely, death. We report a case of adenovirus infection leading to acute liver failure complicated by rhabdomyolysis in a collegiate football player presenting with nausea, vomiting, and diarrhea. We propose a protocol to safely guide the return to play progression for patients with complicated exertional rhabdomyolysis.

## Introduction

Adenoviruses are a common family of viruses that typically infect the upper respiratory and gastrointestinal tracts, leading to conjunctivitis, cough, tonsillitis, and gastroenteritis. It is transmitted by direct contact and fecal-oral transmission with an incubation period of 5-9 days. However, most infections are self-limiting, mild, and require no therapy or only symptomatic treatment [1]. Significant morbidity and mortality from adenovirus illnesses in healthy patients are rare, though in the immunocompromised and neonate/infant population, there have been reports of acute liver failure and death [2-5]. A PubMed database review, however, did not yield any reports of acute liver failure associated with adenovirus infection in a healthy adolescent or young adult.

Exertional rhabdomyolysis (ER) is a condition seen in athletes primarily at the beginning of their training season, in extreme heat, or in the setting of stimulant use, crush injury, or trauma. Dehydration, such as that caused by gastrointestinal illnesses like adenovirus, is also a risk factor for developing ER. Though muscle soreness and damage from vigorous exercise is normal and can be expected, those who develop ER experience a pathologic breakdown of striated muscle fibers leading to the release of myoglobin into the circulation [6,7]. These products of muscle breakdown obstruct the renal tubules leading to renal failure, metabolic acidosis and other electrolyte abnormalities. ER is diagnosed by an increase in serum creatine kinase (CK) levels of at least five times the upper limit of normal or a urine dipstick positive for blood with no or few red blood cells (RBCs) on microscopy in the setting of profound muscle soreness post-activity [8]. Though prior studies have shown clinically significant increases in CK levels among football athletes during training camp, these athletes had normal urinalyses. In contrast, athletes with rhabdomyolysis develop severe muscle pain, swelling or weakness, myoglobinuria, and renal failure [9,10].

Herein we present the case of a previously healthy athletic young adult male who developed acute liver failure and rhabdomyolysis in the setting of adenovirus infection. We also present a novel return to play protocol after ER, developed in conjunction with nephrologists, with graded increases in aerobic and anaerobic activity and utilizing repeat laboratory testing, to determine a patient’s return to sport.

## Case presentation

The patient was a previously healthy 19-year-old male American football offensive lineman with no history of sickle cell trait. He presented to the sports medicine clinic at a National Collegiate Athletic Association (NCAA) Division I undergraduate institution with two days of nausea, occasional vomiting, diarrhea, myalgias, and malaise. Two days prior to clinic presentation, he was several weeks into winter football workouts and felt malaise and chills. At the time, symptoms were thought to be secondary to a lack of adequate pre-workout hydration, and he was removed from further workouts. He had previously completed summer training camp and the fall football season without difficulty. Despite attempts at oral rehydration, he continued to feel malaise, body aches, and had four episodes of non-bloody, non-bilious emesis and two episodes of non-bloody diarrhea. He had traveled to Florida one month prior but denied any other international or wilderness travel. He had several sick contacts with upper respiratory infection (URI) type symptoms. He took 650 mg acetaminophen on the day of symptom onset but denied any other medication, history of alcohol use, or drug ingestions.

At the time of initial medical evaluation, the patient complained of vague upper epigastric discomfort in addition to the symptoms previously described. His vital signs included blood pressure 118/68 mmHg, heart rate 119 beats per minute, temperature 36.3 F, height 6’4”, and weight 283 lbs (BMI 34.4). The exam was notable for mild right upper quadrant tenderness to palpation, faint tonsillar exudate, and anterior cervical lymphadenopathy. He received two liters of intravenous (IV) normal saline, oral ondansetron (Zofran) and had laboratory blood tests sent, consisting of a complete blood count, comprehensive metabolic panel, monospot, and Group A Streptococcus swab. Labs were notable for creatinine 2.0 mg/dL (eGFR 46 mL/min/m^2^), total bilirubin 3.9 mg/dL, aspartate transaminase (AST) 6080 U/L, alanine aminotransferase (ALT) > 3,500 U/L. Given these abnormalities, he was sent to the Emergency Department the same day for further evaluation.

In the Emergency Department, additional labs were notable for international normalized ratio (INR) 5.9, total CK 14,225 U/L, ammonia 84 umol/L and acetaminophen level < 2.0. The non-contrast CT head was negative for bleeding. Urine dipstick showed 2+ ketones and 3+ blood while microscopy showed 0-3 RBCs, consistent with rhabdomyolysis. He was started on N-acetylcysteine (NAC) for liver protection and admitted to the intensive care unit for further monitoring where his AST peaked at 9,655 U/L and his CK immediately trended downwards. Blood PCR testing for cytomegalovirus (CMV), Epstein-Barr virus (EBV), and herpes simplex virus (HSV) came back negative. His hepatitis panel, including hepA immunoglobulin M (IgM), hepB surface antibody, hepC IgM, and hepC immunoglobulin G (IgG), was negative. Right upper quadrant ultrasound showed a liver measuring 17 cm, consistent with hepatomegaly. Multiple sets of blood cultures were negative. His autoimmune work-up, including anti-smooth muscle antibody, anti-nuclear antibody (ANA), liver-kidney-microsome antibody (LKM ab), and mitochondrial antibody were negative. The patient tested positive for Adenovirus DNA PCR and acute infection was confirmed with antibody serum titer ≥ 1:64, indicative of acute infection. After a four-night stay in the hospital and consultation with intensive care, nephrology, infectious disease, hepatology, and liver transplant services, the patient was diagnosed with adenovirus infection leading to ER and acute liver failure. The patient was treated with fresh frozen plasma for his elevated INR, NAC for his acute liver failure, and IV hydration for his rhabdomyolysis. Fortunately, his clinical course was uncomplicated and he was discharged after five days.

To address his return to sport, in consultation with and at the recommendation of a nephrologist, we utilized a 3/3/3 model with a step-wise, three-phase increase in activity in three-week increments. The phases were designed to develop both endurance and strength while minimizing initial muscle strain and breakdown. The patient began this progression six weeks after discharge from the hospital after his labs had all normalized. The patient had a normal creatinine level within three days of hospital admission, CK levels within five days, and liver function tests normalized after six weeks.

During the three weeks of Phase 1, the patient began an aerobic exercise and position-specific drilling (e.g. stationary bike x 20-30 minutes with HR < 120-130 BPM and footwork drills). In the next three weeks, Phase 2, we added anaerobic exercise (e.g. sprints, 150-yard shuttle runs, three-cone technique, exaggerated A-skips) and weights (with an emphasis on repetitions as opposed to maximum intensity), and a goal of full aerobic, but not anaerobic, fitness at the end of this second three-week period. Finally, for the last three weeks, Phase 3, we reintegrated heavy resistance to his training (e.g. sand plyometrics, hill sprints) so that he was “full go” at the end of this progression. Prior to and during each workout, the patient had symptom check-ins with the athletic training staff. At the conclusion of each phase, prior to progressing to the next phase, lab evaluation with CK level, liver function tests, and renal function were checked. He maintained normal labs and remained asymptomatic throughout the progression. After three weeks of full activity, one final lab check was obtained, which was also normal. He went on to be a three-year starter and completed his college career with no further recurrence of symptoms or other significant medical issues.

## Discussion

This case of adenovirus infection complicated by acute liver failure and rhabdomyolysis is particularly challenging for sports medicine physicians, as the large majority of patients in a collegiate athletic setting are young and healthy with illnesses that are self-limiting. Athletes frequently present with nausea, vomiting and diarrhea, myalgias, and malaise, typically secondary to acute gastroenteritis, and are managed with IV rehydration and anti-emetics. However, in this case, the presence of tonsillar exudates, persistent tachycardia, right upper quadrant tenderness, and lymphadenopathy warranted additional workup leading to the diagnosis of liver failure from adenovirus infection and rhabdomyolysis.

After resolution of the acute adenovirus infection, this patient's ongoing clinical course and return to play protocol were designed to ensure full recovery from and prevention of recurrent acute liver failure and rhabdomyolysis. Daily symptom checks with the athletic trainer in conjunction with scheduled serial lab monitoring were used to safely and effectively return this athlete to his normal state of health and athletic performance. There are two prior publications in the literature regarding return to play protocols after rhabdomyolysis [8,11]. We built upon these previous recommendations and sought to further develop a concrete, multi-disciplinary based return progression with training, clinical, and laboratory goals. Herein we proposed a return to play protocol for athletes with rhabdomyolysis (Table 1). Our proposed protocol, developed in conjunction with nephrology, allows for gradual building of fitness and conditioning in a manner that would safely return the player to full fitness within six weeks of return and full competition within nine weeks. If an athlete is unable to progress through the return to play phases due to recurrent elevations in serum creatinine or CK levels, ongoing exercise should cease and further investigation and a nephrology consultation should be considered.

**Table 1:** Return to play algorithm after rhabdomyolysis Consider pre/post workout weights and urine specific gravity checks if available to ensure proper hydration before each bout of exercise. *Creatine kinase (CK) level, liver function tests (LFTs), and renal function should be checked and at the end of each phase before advancing. **If unable to progress in RTP algorithm due to ongoing lab abnormalities, consider nephrology referral. ***Labs should be re-checked 2-3 weeks after completion of phase 3. HR - heart rate

	Activity	Examples:	End-Phase Goal	Minimum duration*
Phase 0	Activities of daily living and ensuring normal sleep	N/A	Normalization of lab abnormalities**	2 weeks
Phase 1	Aerobic exercise and position-specific drilling. Max HR < 120-130 bpm	Stationary bike, incline treadmill x 20-30 minutes	Initiate basic fitness activity	3 weeks
Phase 2	Anaerobic exercise and weight lifting	Shuttle runs, sled work	Full aerobic fitness	3 weeks
Phase 3	Heavy resistance training	Sand plyometrics, hill sprints	Cleared for full activity***	3 weeks

We report a unique case of acute liver failure and rhabdomyolysis in a young healthy collegiate male athlete presumed to be secondary to adenovirus infection. Though there have been previous case reports of adenovirus leading to acute liver failure in toddlers [2,5] and in the elderly immunocompromised population [3,4,12], this is the first reported case of adenovirus associated liver failure in a healthy, young adult. Although rhabdomyolysis has been linked to elevated liver aminotransferases with peak ALT/AST levels around 600 U/L [13,14], it has never been shown to lead to acute liver failure with profound elevations in liver aminotransferases, INR and splenomegaly as in this case. This case is also atypical in that this occurred near the end of the winter conditioning period when many of the typical risk factors for rhabdomyolysis such as lack of acclimatization, overexertion, and extreme heat are not present. However, the concurrent adenovirus infection may have been a “second hit,” which caused the patient to progress into liver failure.

## Conclusions

ER is a well-known condition in sports medicine leading to multi-organ failure. However, there have been few studies looking at returning athletes to play after being treated for this ailment. We propose a graded return to play progression that can be used to guide the clinician in returning a recovering athlete to full participation after ER. Further studies are necessary to establish the long-term safety and generalizability of this return to play progression post-ER.
